# Aerobic Interval Training and Cardiometabolic Health in Patients with Type 2 Diabetes: A Meta-Analysis

**DOI:** 10.3389/fphys.2017.00957

**Published:** 2017-11-23

**Authors:** Shanhu Qiu, Xue Cai, Zilin Sun, Martina Zügel, Jürgen M. Steinacker, Uwe Schumann

**Affiliations:** ^1^Department of Endocrinology, Institute of Diabetes, School of Medicine, Zhongda Hospital, Southeast University, Nanjing, China; ^2^Division of Sports and Rehabilitation Medicine, Ulm University Medical Center, Ulm, Germany

**Keywords:** aerobic interval training, moderate-intensity continuous training, cardiorespiratory fitness, glycemic control, type 2 diabetes

## Abstract

Vigorous to maximal aerobic interval training (INT) has received remarkable interest in improving cardiometabolic outcomes for type 2 diabetes patients recently, yet with inconsistent findings. This meta-analysis was aimed to quantify its effectiveness in type 2 diabetes. Randomized controlled trials (RCTs) were identified by searches of 3 databases to October 2017, which evaluated the effects of INT with a minimal training duration of 8 weeks vs. moderate-intensity continuous training (MICT) or non-exercise training (NET) among type 2 diabetes patients on outcomes including cardiorespiratory fitness, glycemic control, body composition, blood pressure, and lipid profiles. Weighted mean differences with 95% confidence intervals (CIs) were calculated with the random-effects model. Nine datasets from 7 RCTs with 189 patients were included. Compared with MICT, INT improved maximal oxygen consumption (VO_2max_) by 2.60 ml/kg/min (95% CI: 1.32 to 3.88 ml/kg/min, *P* <0.001) and decreased hemoglobin A1c (HbA1c) by 0.26% (95% CI: −0.46% to −0.07%, *P* = 0.008). These outcomes for INT were also significant vs. energy expenditure-matched MICT, with VO_2max_ increased by 2.18 ml/kg/min (*P* = 0.04) and HbA1c decreased by 0.28% (*P* = 0.01). Yet their magnitudes of changes were larger compared with NET, with VO_2max_ increased by 6.38 ml/kg/min (*P* <0.001) and HbA1c reduced by 0.83% (*P* = 0.004). Systolic blood pressure could be lowered by INT compared with energy expenditure-matched MICT or NET (both *P* <0.05), but other cardiometabolic markers and body composition were not significantly altered in general. In conclusion, despite a limited number of studies, INT improves cardiometabolic health especially for VO_2max_ and HbA1c among patients with type 2 diabetes, and might be considered an alternative to MICT. Yet the optimal training protocols still require to be established.

## Introduction

Exercise training has long been considered a key element in the management of type 2 diabetes (Colberg et al., 2010; Qiu et al., 2014). Recent practice guidelines advise patients with type 2 diabetes to engage in at least 150 min per week of moderate-to-vigorous aerobic exercise sustained in bouts lasting at least 10 min and spread throughout the week, in order to achieve optimal glycemic control and some other cardiometabolic benefits such as improved cardiorespiratory fitness and lowered blood pressure (Colberg et al., 2010). The existing recommendation has a primary emphasis on continuous aerobic exercise, yet it still remains uncertain whether it is the best form of exercise for patients with type 2 diabetes, since epidemiological evidence shows that they are less likely to undertake physical activity at recommended levels compared with those without diabetes (Zhao et al., 2011).

Different from continuous aerobic exercise, aerobic interval training is a form of exercise that is characterized by repeated short bursts of aerobic exercise at an intense intensity (e.g., vigorous- or high-intensity) interspersed with brief periods of complete rest (passive recovery) or low-to-moderate intensity exercise (active recovery) (Gibala et al., 2012; Pattyn et al., 2014; Milanovic et al., 2015; Stöggl and Björklund, 2017). Emerging evidence suggests that vigorous to maximal aerobic interval training (INT) (Haykowsky et al., 2013), is able to produce favorable cardiorespiratory or metabolic benefits to a similar or even larger extent compared with moderate-intensity continuous training (MICT) among overweight and obese youth (Garcia-Hermoso et al., 2016) or in patients with chronic heart failure (Haykowsky et al., 2013; Smart et al., 2013; Garcia-Hermoso et al., 2016), indicating that INT might be an alternative to continuous training. In recent years a growing attention has been attracted to use this form of training in patients with type 2 diabetes (Karstoft et al., 2013; Terada et al., 2013; Hollekim-Strand et al., 2014; Mitranun et al., 2014; Taylor et al., 2014; Lee et al., 2015; Alvarez et al., 2016; Cassidy et al., 2016; Maillard et al., 2016; Bellia et al., 2017; Francois et al., 2017). However, results from individual studies remain inconsistent (Karstoft et al., 2013; Terada et al., 2013; Hollekim-Strand et al., 2014; Mitranun et al., 2014; Alvarez et al., 2016; Cassidy et al., 2016; Maillard et al., 2016). For example, the study by Terada et al. showed that INT had a minor effect in increasing maximal oxygen consumption (VO_2max_) but a marginal effect in decreasing glycosylated hemoglobin A1c (HbA1c) compared with MICT (Terada et al., 2013). Conversely, the others suggested that INT may significantly increase VO_2max_ (Karstoft et al., 2013; Hollekim-Strand et al., 2014), but had limited power in decreasing HbA1c (Karstoft et al., 2013; Hollekim-Strand et al., 2014). Moreover, the sample sizes of all individual studies were rather small, which ranged from 15 to 37 (Karstoft et al., 2013; Terada et al., 2013; Hollekim-Strand et al., 2014; Mitranun et al., 2014; Alvarez et al., 2016; Cassidy et al., 2016; Maillard et al., 2016).

There were several narrative reviews and meta-analyses that had been performed recently with an attempt to assess the impact of INT for patients with type 2 diabetes (Jelleyman et al., 2015; Hamasaki, 2016; Liubaoerjijin et al., 2016; Cassidy et al., 2017). However, some did not apply systematic literature searching strategies nor did they provide quantitative results (Hamasaki, 2016; Cassidy et al., 2017), while others focused on glycemic control or enrolled heterogeneous populations other than type 2 diabetes only (Jelleyman et al., 2015; Liubaoerjijin et al., 2016). Noteworthy, none of them had comprehensively evaluated the efficacy of INT vs. MICT or non-exercise training (NET) on other cardiometabolic markers such as blood pressure or lipid profiles (Jelleyman et al., 2015; Hamasaki, 2016; Liubaoerjijin et al., 2016; Cassidy et al., 2017), both of which are considered the mainstays of type 2 diabetes management (Colberg et al., 2010).

In order to facilitate the understanding of the training effects of INT, the present meta-analysis of randomized controlled trials (RCTs) was aimed to quantify its effectiveness in improving cardiometabolic health including cardiorespiratory fitness, glycemic control, body composition, blood pressure, and lipid profiles, as compared with MICT and NET among patients with type 2 diabetes.

## Materials and methods

### Literature search and selection

An extensive literature search with a language restriction to English was performed in databases of PubMed, Web of Science, and Cochrane Library up to October 18th, 2017, using the terms or words related to INT (that is, “interval training” or “interval exercise” or “intermittent exercise” or “intermittent training” or “sprint exercise” or “sprint training” or “circuit training” or “circuit exercise” or “high intensity exercise”) and diabetes (that is, “diabetes” or “diabetes mellitus” or “diabetic” or “diabetics”). In addition, a manual check of the reference lists of related reviews and eligible articles was performed for other appropriate studies. This meta-analysis was conducted in accordance with the Preferred Reporting Items for Systematic Reviews and Meta-Analyses (PRISMA) guideline, and was prospectively registered in PROSPERO as CRD 42017055972.

### Study selection

Studies were included if they: (1) enrolled only patients with type 2 diabetes, (2) received INT interventions and compared with MICT or NET, (3) were RCTs, and (4) reported any of the following data related to cardiometabolic health: cardiorespiratory fitness (indicated by VO_2max_), glycemic control (assessed by HbA1c), body composition [evaluated by body weight, body mass index (BMI), or fat mass], blood pressure [determined by systolic blood pressure (SBP) or diastolic blood pressure (DBP)], or lipid profiles [measured by triglycerides (TG), total cholesterol (TC), high-density lipoprotein cholesterol (HDL-C), or low-density lipoprotein cholesterol (LDL-C)].

In this meta-analysis the exercise intensity for INT was referred to vigorous- or high-intensity defined according to the position statement on physical activity and exercise intensity from Norton et al. (2010). Moreover, it is expected that 8–12 weeks of exercise training may confer some benefits to glycemic and weight control (Boulé et al., 2001; Chudyk and Petrella, 2011; Qiu et al., 2014), and given that HbA1c reflects average blood glucose level in the past 8–12 weeks, studies for inclusion were therefore restricted to those with minimal training durations of 8 weeks.

Studies that did not meet the above inclusion criteria or that had overlaps in populations (that is, enrolling the same populations from the same center) were excluded.

## Data collection and quality assessment

The following data were collected from each individual study: first author, publication year, geographic location, severity of disease, numbers of participants, proportions of males, baseline mean ages, mean BMI, time since diagnose of diabetes (that is, duration of diabetes), periods of intervention, protocols of INT and MICT such as intensity, time per bout, and recovery periods, outcomes of interest, adherence rates to INT, dropout rates, and adverse events like hypoglycemia, exercise-related injuries, or cardiac arrests. During the data collection, data on the outcome of HbA1c from the study by Alvarez et al. were obtained from the corresponding author (Alvarez et al., 2016).

The quality of each included study was assessed with reference to the Cochrane Collaboration “Risk of Bias” tool (Higgins et al., 2008; Wang et al., 2016), which focuses mainly on items related to selection bias, performance bias, detection bias, attrition bias, and reporting bias. All data collection and quality assessment were conducted initially by one author, and were further cross-checked by another one. Discrepancies were resolved by discussion with a third author.

### Statistical analysis

The weighted mean difference (WMD) was chosen as the main effect size for all outcomes of interest except fat mass, for which the standard mean difference (SMD) was selected due to the different measuring scales. Post-intervention mean values or change scores with the corresponding standard deviations (SDs) from eligible studies were entered into the meta-analyses using a random-effects model to get the summary effect sizes (Higgins et al., 2008), which were considered statistically significant at *P* <0.05. In cases that both post-intervention mean values and change scores were provided, changes scores were primarily used. When studies reported the standard error of a mean, the SDs were calculated with reference to the formula proposed previously (Higgins et al., 2008). Because of the different control groups (MICT and NET), separate meta-analyses were conducted accordingly. Heterogeneity was assessed using the *I*^2^ statistic, with the value >50% indicative of statistical heterogeneity (Higgins et al., 2008).

Sensitivity analyses were performed to assess the effects of INT vs. energy expenditure-matched MICT on cardiometabolic factors. Univariate meta-regression analyses were employed to determine the associations of age (logarithmically transformed), BMI, duration of diabetes, HbA1c, and VO_2max_ at baseline as well as training duration with changes in all outcomes of interest. Results for meta-regression analyses were considered significant at *P* <0.05. Publication bias was analyzed using the Egger test, with *P* <0.10 considered significant. All the above statistical analyses were conducted using STATA software (version 12.0; College Station, Texas, USA).

## Results

### Search results and study characteristics

**Figure 1:**
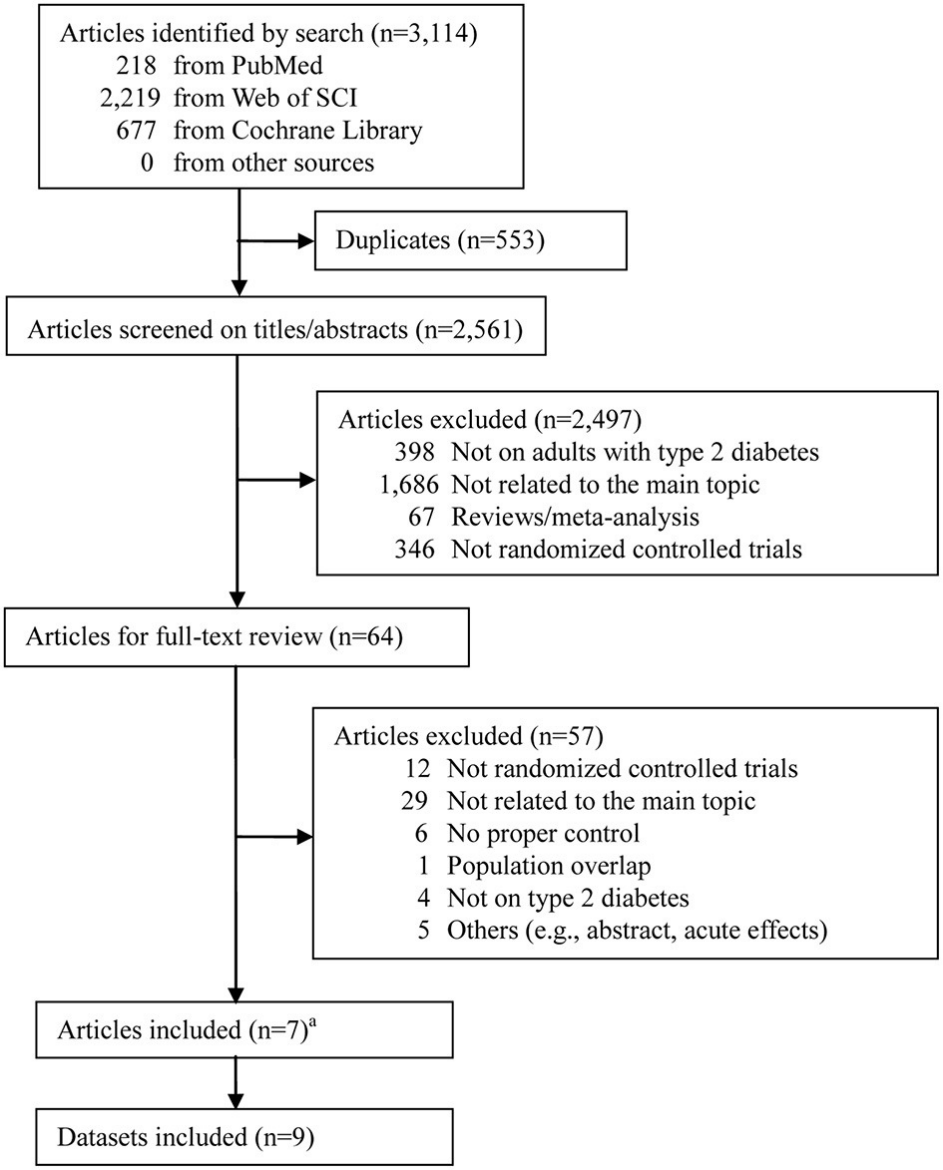
Literature search flow. ^a^ Two articles Karstoft et al. (2013) and Mitranun et al. (2014) had 2 control groups each.

The process of literature search and study selection is shown in Figure 1. The initial search strategy yielded 3,114 published articles in total (218 from PubMed, 677 from Cochrane Library, and 2,219 from Web of Science). Among them, 7 RCTs were identified to be eligible for this meta-analysis after removing duplicates, screening on titles/abstracts, and full-text reviewing (Karstoft et al., 2013; Terada et al., 2013; Hollekim-Strand et al., 2014; Mitranun et al., 2014; Alvarez et al., 2016; Cassidy et al., 2016; Maillard et al., 2016). Notably, among the excluded studies, the one by Bellia et al. (2017) was excluded because the control group was asked to undertake unstructured and unsupervised physical activity, which therefore cannot be identified as MICT or NET. Moreover, since 2 RCTs had 2 different control groups each (Karstoft et al., 2013; Mitranun et al., 2014), a total of 9 datasets were finally included.

The characteristics of those included studies are presented in Table 1. A total of 189 clinically stable patients with type 2 diabetes were enrolled, with 86 of them randomized to INT, 59 to MICT, and 44 to NET. The mean age of all these patients was 58.8 (SD = 7.5) years, and their mean BMI was 30.4 (SD = 0.7) kg/m^2^. The average duration of diabetes for these patients was 8.3 (SD = 6.6) years. More than half of the enrolled patients took antihyperglycemic medications, with metformin as the most commonly used one; but they were instructed not to change their dosages throughout the interventions in general.

**Table 1:** Characteristics of the included randomized controlled studies.

**Source**	**Origin**	**Age (year)/Gender^a^**	**INT interventions^b^**	**Control interventions**
**INT vs. MICT**				
Karstoft et al., 2013	Denmark	59.2; 62.5%	10 × (3-min walking at about 90% PEER^c^, 3-min walking at about 54% PEER); 5 times/week, 16 weeks	60 min of walking at about 73% PEER each time, 5 times/week, 16 weeks
Terada et al., 2013	Canada	63; 53.3%	7–15 × (1-min cycling or walking at 100% VO_2_R, 3-min cycling or walking at 40%VO_2_R); 5 times/week, 12 weeks	30–60 min of cycling or walking at 40% VO_2_R each time, 5 times/week, 12 weeks
Mitranun et al., 2014	Thailand	61.5; 35.7%	20 min walking or running at 50% VO_2peak_ for phase 1; 4 × (1-min walking or running at 80% VO_2peak_, 4-min walking or running at 50% VO_2peak_) for phase 2; and 6 × (1-min walking or running at 85% VO_2peak_, 4-min walking or running at 60% VO_2peak_) for phase 3; 3 times/week, 12 weeks in total	20 min walking or running at 50% VO_2peak_ for phase 1; 20-min walking or running at 60% VO_2peak_ for phase 2; and 30-min walking or running at 65% VO_2peak_ for phase 3; 3 times/week, 12 weeks in total
Hollekim-Strand et al., 2014	Norway	55.9; 64.0%	4 × (4-min walking or jogging at 90 to 95% HR_max_, 3-min walking or jogging at 70% HR_max_); 3 times/week, 12 weeks	210 min of home-based moderate intensity exercise every week, 12 weeks
Maillard et al., 2016	France	69; 0	60 × (8-sec cycling at supramaximal intensity, 12-s of slow cycling); 2 times/week, 16 weeks	40 min of cycling at 55–60% HR_reserve_ each time, 2 times/week, 16 weeks
**INT vs. NET**				
Karstoft et al., 2013	Denmark	57.3; 60.0%	10 × (3-min walking at about 90% PEER^c^, 3-min walking at about 54% PEER); 5 times/week, 16 weeks	Instructed to continue the habitual lifestyle, 16 weeks
Mitranun et al., 2014	Thailand	61.5; 34.5%	20 min walking or running at 50% VO_2peak_ for phase 1; 4 × (1-min walking or running at 80% VO_2peak_, 4-min walking or running at 50% VO_2peak_) for phase 2; 6 × (1-min walking or running at 85% VO_2peak_, 4-min walking or running at 60% VO_2peak_) for phase 3; 3 times/week, 12 weeks in total	Instructed to remain sedentary as they previously were, 12 weeks
Alvarez et al., 2016	Brazil	44.5; 0	8–14 × (0.5- to 1-min jogging or running at 90–100% HR_reserve_, 1.6- to 2-min walking at ≤ 70% HR_reserve_); 3 times/week, 12 weeks	Instructed to continue the habitual lifestyle, 16 weeks
Cassidy et al., 2016	UK	60; 78.3%	5 × (2- to 3.8-min cycling at RPE 16–17, 3-min passive and light recovery); 3 times/week, 12 weeks	Instructed to continue the habitual lifestyle and care, 12 weeks

*INT, vigorous to maximal aerobic interval training; MICT, moderate-intensity continuous training; PEER, peak energyexpenditure rate; VO_2_R, oxygen consumption reserve; VO_2peak_, oxygen consumption peak; HR_max_, maximum heart rate; HR_reserve_, heart rate reserve; NET, non-exercise training; RPE, Rating of perceived exertion*.

a *Gender here represents the proportions of men*.

b *For the details of each session of INT, they were expressed as number of intervals x (details of the high-intensity exercise, details of the active or passive recovery)*.

c *Exercise intensity was measured by a JD Mate during walking*.

Of the eligible studies, the reported training modalities of INT included cycling, walking, jogging, and running. The frequency of INT ranged from 2 to 5 times per week, with most of them utilizing 3 or 5 times per week. The exercise intensity of INT was provided in terms of different but qualified scales including VO_2_ peak, VO_2_ reserve, peak energy-expenditure rate, maximum heart rate, heart rate reserve, and Borg Rating of Perceived Exertion. For every INT session that excludes the warm-up and cool-down sessions but includes the recovery period during each interval of prescribed vigorous to maximal exercise, its total length ranged from 20 to 60 min (with each interval of INT lasting mostly for 1 to 4 min). The training duration of INT was reported to be 12 or 16 weeks (Table 1).

For the 5 studies that used MICT in comparison with INT, the frequency varied from 2 to 5 times, the intensity ranged from 40 to 65% of the corresponding scale aforementioned, and the length differed from 60 to 300 min per week. For the patients in the 4 studies that received no exercise training, they were all advised to continue their habitual lifestyle (Table 1).

No cardiovascular events or sport-related injuries associated with INT were reported (Terada et al., 2013; Alvarez et al., 2016; Cassidy et al., 2016; Maillard et al., 2016), but one study observed the new-onset asthmatic symptoms in one patient during INT (Karstoft et al., 2013). Additionally, that study also reported knee injury in one patient during MICT (Karstoft et al., 2013). The adherence to INT intervention was high, with a mean result over 90% in studies with available data (Karstoft et al., 2013; Terada et al., 2013; Hollekim-Strand et al., 2014; Alvarez et al., 2016; Cassidy et al., 2016), and this adherence was found to be comparable to that of MICT (Terada et al., 2013; Hollekim-Strand et al., 2014). The study quality of each included RCT was generally fair (Table 2).

**Table 2:** Bias assessment of each randomized controlled trial.

**Source**	**Random sequence generation**	**Allocation concealment**	**Blinding of participants and personnel**	**Blinding of outcome assessment**	**Incomplete outcome data addressed**	**Selective reporting**
Karstoft et al., 2013	Low	Unclear	Low	Low	Low	Low
Terada et al., 2013	Low	Unclear	Low	Low	Low	Low
Mitranun et al., 2014	Unclear	Unclear	Low	Low	Low	Low
Hollekim-Strand et al., 2014	Low	Unclear	Low	Low	High	Low
Maillard et al., 2016	Low	Unclear	Low	Low	Low	Low
Alvarez et al., 2016	Low	Unclear	Low	Low	High	Low
Cassidy et al., 2016	Low	Unclear	Low	Low	Low	Low

### INT and cardiorespiratory fitness

**Figure 2:**
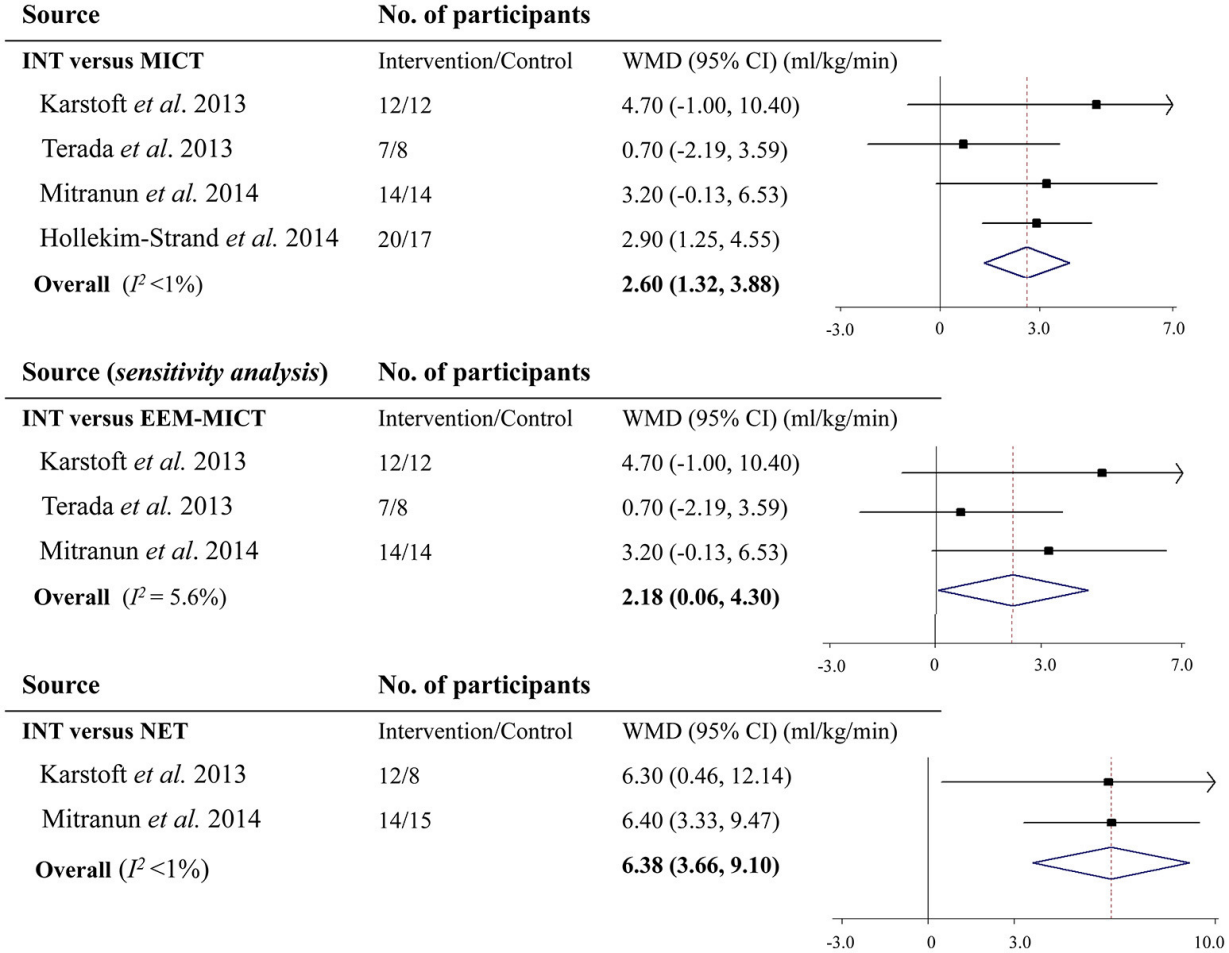
Effects of vigorous to maximal aerobic interval training on maximal oxygen consumption in patients with type 2 diabetes. INT, vigorous to maximal aerobic interval training; MICT, moderate-intensity continuous training; NET, non-exercise training; WMD, weighted mean difference; CI, confidence interval; EEM-MICT, energy expenditure-matched moderate-intensity continuous training.

Of the 4 studies that reported the effect of INT on cardiorespiratory fitness assessed by VO_2max_ vs. MICT, only the one by Hollekim-Strand et al. (2014) observed a significant improvement. Pooled results from the meta-analysis showed that compared with MICT, INT increased VO_2max_ by 2.60 ml/kg/min (95% CI: 1.32 to 3.88 ml/kg/min, *P* <0.001; *I*^2^ <1%; Figure 2). When restricting studies to those, which matched energy expenditure, INT still increased VO_2max_ over MICT by 2.18 ml/kg/min (95% CI: 0.06 to 4.30 ml/kg/min, *P* = 0.04; *I*^2^ = 5.6%; Figure 2). Moreover, upon removal of each individual study at one time, all the re-analyzed results were statistically significant (all *P* <0.05) and remained largely unchanged.

There were 2 studies reporting the INT effect on VO_2max_ in comparison with NET. Meta-analysis showed that VO_2max_ was increased by 6.38 ml/kg/min (95% CI: 3.66 to 9.10 ml/kg/min, *P* <0.001; *I*^2^ <1%; Figure 2).

### INT and glycemic control

**Figure 3:**
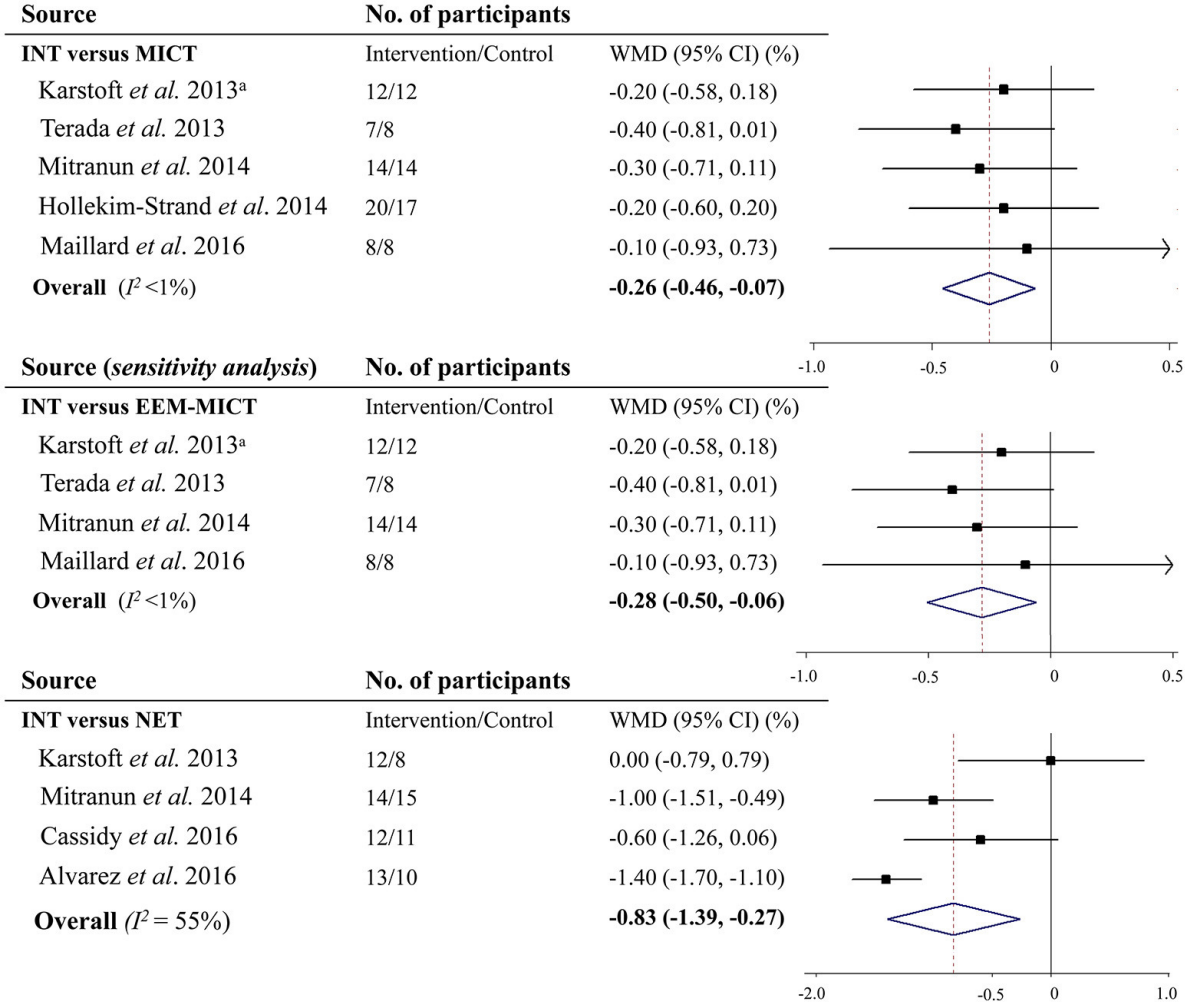
Effects of vigorous to maximal aerobic interval training on glycosylated hemoglobin A1c in patients with type 2 diabetes. INT, vigorous to maximal aerobic interval training; MICT, moderate-intensity continuous training; NET, non-exercise training; WMD, weighted mean difference; CI, confidence interval; EEM-MICT, energy expenditure-matched moderate-intensity continuous training. ^a^Data were obtained from the study by Liubaoerjijin et al. (2016).

Five studies compared INT with MICT and provided results on glycemic control as measured by HbA1c. And 4 of them showed that INT did not decrease HbA1c over MICT. However, by pooling all 5 studies together, results suggested that INT significantly decreased HbA1c by 0.26% [2.8 mmol/mol] (95% CI: −0.46 to −0.07% [−5.0 to −0.8 mmol/mol], *P* = 0.008; *I*^2^ <1%) in comparison with MICT (Figure 3). Sensitivity analysis showed that the decrease in HbA1c by INT vs. the energy expenditure-matched MICT (WMD −0.28% [−3.1 mmol/mol], 95% CI: −0.50 to −0.06% [−5.5 to −0.7 mmol/mol], *I*^2^ <1%) was also statistically significant (*P* = 0.01; Figure 3). Moreover, when removing a single study at one time, all the re-analyzed results were significant (all *P* <0.05), resulting in minor changes only.

Four studies reported results on HbA1c comparing INT with NET. Subsequent meta-analysis showed that INT led to a decrease in HbA1c by 0.83% (9.1 mmol/mol) (95% CI: −1.39% to −0.27% [−15.2 to −3.0 mmol/mol], *P* = 0.004; *I*^2^ = 78.1%) vs. NET (Figure 3).

### INT and body composition

For INT vs. MICT, there were 5, 4, and 5 studies enrolled in the meta-analysis, respectively for BMI, body weight, and fat mass (Table 3). Results showed that INT was not associated with any significant change in BMI (WMD −0.16 kg/m^2^, 95% CI: −0.57 to 0.24 kg/m^2^, *P* = 0.43; *I*^2^ <1%), body weight (WMD 0.39 kg, 95% CI: −1.33 to 2.11 kg, *P* = 0.66; *I*^2^ <1%), or body fat mass (SMD −0.15, 95% CI: −0.51 to 0.21, *P* = 0.40; *I*^2^ <1%) compared with MICT. There were also no changes on these outcomes in relation to INT (*P* = 0.79 for BMI, 0.66 for body weight, and 0.31 for body fat mass) over energy expenditure-matched MICT.

**Table 3:** Effects of vigorous to maximal aerobic interval training on other cardiometabolic factors in type 2 diabetes patients^a^.

**Outcomes**	**INT vs. MICT**		**INT vs. energy expenditure-matched MICT**		**INT vs. NET**	
	**No**.	**WMD (95% CI)**	**No**.	**WMD (95% CI)**	**No**.	**WMD (95% CI)**
**BODY COMPOSITION**						
BMI (kg/m^2^)	5	−0.16 (−0.57 to 0.24)	4	−0.13 (−1.06 to 0.81)	3	−0.90 (−2.00 to 0.21)
Weight (kg)	4	0.39 (−1.33 to 2.11)	4	0.39 (−1.33 to 2.11)	4	−3.36 (−7.24 to 0.52)
Fat mass^b^	5	−0.15 (−0.51 to 0.21)^b^	4	−0.22 (−0.66 to 0.21)^b^	3	−0.49 (−0.96 to −0.01)^b^^c^
**BLOOD PRESSURE**						
SBP (mmHg)	3	−7.07 (−17.31 to 3.17)	2	−11.96 (−23.30 to −0.63)^c^	4	−2.23 (−4.37 to −0.10)^c^
DBP (mmHg)	3	−2.40 (−5.71 to 0.91)	2	−1.66 (−6.13 to 2.81)	4	−0.64 (−2.00 to 0.71)
**LIPID PROFILES**						
TG (mmol/L)	4	0.40 (−0.18 to 0.97)	4	0.40 (−0.18 to 0.97)	4	−0.22 (−0.47 to 0.03)
TC (mmol/L)	4	−0.11 (−0.51 to 0.30)	4	−0.11 (−0.51 to 0.30)	4	−0.64 (−1.05 to −0.23)^c^
HDL-C (mmol/L)	4	−0.11 (−0.24 to 0.03)	4	−0.11 (−0.24 to 0.03)	3	0.20 (−0.08 to 0.47)
LDL-C (mmol/L)	4	−0.09 (−0.52 to 0.35)	4	−0.09 (−0.52 to 0.35)	3	−0.55 (−1.01 to −0.09)^c^

*INT, vigorous to maximal aerobic interval training; MICT, moderate-intensity continuous training; NET, non-exercise training; WMD, weighted mean difference; CI, confidence interval; BMI, body mass index; SBP, systolic blood pressure; DBP, diastolic blood pressure; TG, triglycerides; TC, total cholesterol; HDL-C, high-density lipoprotein cholesterol; LDL-C, low-density lipoprotein cholesterol*.

a *All effect sizes were calculated using a random-effects meta-analysis model*.

b *Standard mean difference was chosen because of the different measuring scales*.

c *P was less than 0.05, indicative of statistical significance*.

For INT vs. NET, the summary effect sizes for BMI (3 studies; WMD −0.90 kg/m^2^, 95% CI: −2.00 to 0.21 kg/m^2^, *P* = 0.11; *I*^2^ <1%) and body weight (4 studies, WMD −3.36 kg, 95% CI: −7.24 to 0.52 kg, *P* = 0.09; *I*^2^ <1%) were not statistically significant, except for body fat mass (3 studies, SMD −0.49, 95% CI: −0.96 to −0.01, *P* = 0.04; *I*^2^ <1%; Table 3).

### INT and blood pressure

For results on blood pressure, 3 studies made comparisons between INT and MICT, and 4 between INT and NET (Table 3). Meta-analyses showed that INT did not reduce SBP vs. MICT (WMD −7.07 mmHg, 95% CI: −17.31 to 3.17 mmHg, *P* = 0.18; *I*^2^ = 53.8%) but lowered SBP vs. NET (WMD −2.23 mmHg, 95% CI: −4.37 to −0.10 mmHg, *P* = 0.04; *I*^2^ <1%). There were no significant changes on DBP associated with INT compared with neither MICT nor NET (WMD −2.40 mmHg, 95% CI: −5.71 to 0.91 mmHg, *P* = 0.16; *I*^2^ <1%; and WMD −0.64 mmHg, 95% CI: −2.00 to 0.71 mmHg, *P* = 0.35; *I*^2^ <1%; respectively). When compared with energy expenditure-matched MICT, INT significantly reduced SBP (2 studies, WMD −11.96 mmHg, 95% CI: −23.30 to −0.63 mmHg, *P* = 0.04; *I*^2^ = 24.8%), but not DBP (2 studies, WMD −1.66 mmHg, 95% CI: −6.13 to 2.81 mmHg, *P* = 0.48; *I*^2^ <1%).

### INT and lipid profiles

Four studies compared energy expenditure-matched INT with MICT and reported its effect on lipid profiles (Table 3). Pooled results showed that INT did not alter the lipid profiles including TG (WMD 0.40 mmol/L, 95% CI: −0.18 to 0.97 mmol/L, *P* = 0.18; *I*^2^ = 33.0%), TC (WMD −0.11 mmol/L, 95% CI: −0.51 to 0.30 mmol/L, *P* = 0.61; *I*^2^ = 31.5%), HDL-C (WMD −0.11 mmol/L, 95% CI: −0.24 to 0.03 mmol/L, *P* = 0.12; *I*^2^ = 52.2%), and LDL-C (WMD −0.09 mmol/L, 95% CI: −0.52 to 0.35 mmol/L, *P* = 0.70; *I*^2^ = 68.5%) over MICT, but it significantly reduced TC (4 studies, WMD −0.64 mmol/L, 95% CI: −1.05 to −0.23 mmol/L, *P* = 0.002; *I*^2^ = 47.2%) over NET.

### Meta-regression analysis and publication bias

Univariate meta-regression analyses showed that the effects of INT vs. MICT or NET on cardiometabolic factors were not significantly moderated by any of the variables described in “Materials and Methods” part (Table 4). There was no evidence of publication bias using Egger's test for most of the cardiometabolic factors (All *P* > 0.10) except fat mass (*P* = 0.02 in the category of INT vs. MICT) and HbA1c (*P* = 0.09 in the category of INT vs. NET).

**Table 4:** Meta-regression analysis of outcomes of interest.

	**Beta-coefficient (*****P*****)**					
	**Age^a^**	**BMI**	**Diabetes duration**	**HbA1c**	**VO_2max_**	**Training duration**
**INT vs. MICT**						
VO_2max_	−11.3 (0.47)	−0.30 (0.28)	0.02 (0.91)	1.24 (0.62)	0.12 (0.44)	0.55 (0.54)
HbA1c	−0.59 (0.77)	−0.02 (0.53)	−0.003 (0.85)	0.008 (0.98)	0.02 (0.56)	0.03 (0.63)
BMI	3.07 (0.55)	0.06 (0.40)	−0.01 (0.78)	−0.50 (0.59)	−0.02 (0.79)	0.07 (0.87)
Weight	69.03 (0.33)	0.22 (0.65)	0.05 (0.90)	0.47 (0.92)	−0.89 (0.48)	−0.34 (0.79)
Fat mass^b^	−1.67 (0.58)	−0.02 (0.77)	−0.003 (0.91)	0.006 (0.99)	0.02 (0.71)	−0.05 (0.62)
SBP	−97.60 (0.65)	23.92 (0.39)	0.007 (0.99)	4.69 (0.83)	1.25 (0.59)	−4.09 (0.31)
DBP	30.17 (0.58)	−3.28 (0.71)	0.23 (0.49)	4.41 (0.47)	−0.31 (0.61)	−0.71 (0.63)
TG	4.82 (0.49)	0.04 (0.71)	−0.07 (0.24)	−0.66 (0.42)	0.003 (0.99)	0.22 (0.24)
TC	7.56 (0.22)	0.07 (0.25)	0.01 (0.89)	0.07 (0.91)	−0.15 (0.31)	−0.06 (0.67)
HDL-C	0.40 (0.87)	0.02 (0.36)	0.003 (0.85)	−0.002 (0.99)	−0.04 (0.28)	−0.07 (0.14)
LDL-C	7.69 (0.10)	0.06 (0.45)	0.02 (0.76)	0.24 (0.73)	−0.13 (0.26)	−0.02 (0.90)
**INT vs. NET**						
VO_2max_	NA	NA	NA	NA	NA	NA
HbA1c	2.34 (0.37)	−0.08 (0.84)	−0.01 (0.81)	−0.77 (0.51)	0.07 (0.78)	0.001 (0.99)
BMI	−1.32 (0.81)	0.78 (0.71)	−0.002 (0.99)	0.24 (0.92)	NA	0.004 (0.99)
Weight	−1.07 (0.94)	1.31 (0.68)	0.05 (0.89)	2.76 (0.75)	−1.02 (0.66)	−0.35 (0.77)
Fat mass^b^	−3.84 (0.75)	−0.009 (0.97)	−0.01 (0.78)	−0.24 (0.76)	0.02 (0.93)	NA
SBP^b^	0.40 (0.83)	−0.02 (0.93)	−0.004 (0.89)	−0.18 (0.79)	0.02 (0.90)	0.01 (0.93)
DBP	6.28 (0.36)	1.16 (0.30)	0.04 (0.89)	−0.35 (0.95)	−0.64 (0.54)	−0.54 (0.30)
TG	1.00 (0.23)	0.16 (0.35)	−0.005 (0.86)	−0.35 (0.61)	−0.03 (0.84)	−0.07 (0.26)
TC	−1.09 (0.57)	0.42 (0.16)	−0.32 (0.24)	−0.53 (0.49)	−0.23 (0.32)	0.001 (0.99)
HDL-C	−1.25 (0.59)	0.51 (0.33)	0.006 (0.91)	0.38 (0.69)	NA	−0.03 (0.89)
LDL-C	−1.95 (0.26)	0.69 (0.25)	−0.02 (0.73)	−0.05 (0.96)	NA	0.06 (0.74)

*BMI, body mass index; HbA1c, glycosylated hemoglobin A1c; VO_2max_, maximal oxygen consumption; INT, vigorous to maximal aerobic interval training; MICT, moderate-intensity continuous training; SBP, systolic blood pressure; DBP, diastolic blood pressure; TG, triglycerides; TC, total cholesterol; HDL-C, high-density lipoprotein cholesterol; LDL-C, low-density lipoprotein cholesterol; NET, non-exercise training; NA, not applicable*.

a *Age data were log-transformed for analysis*.

b *Standard mean difference was chosen because of the different measuring scales*.

## Discussion

### Main findings

This meta-analysis showed that INT is effective in improving cardiorespiratory fitness and glycemic control compared with MICT or NET in clinically stable patients with type 2 diabetes, and such effects are not likely to be affected by baseline age, BMI, or fitness levels assessed by HbA1c or VO_2max_. INT may also reduce SBP, but the evidence for its effectiveness in altering body composition, lowering DBP, and changing lipid profiles in comparison with MICT or NET remains inconclusive in general.

### Interpretations

Several systematic reviews or meta-analyses noted that INT is a highly effective approach in improving cardiorespiratory fitness not only in overweight and obese youth (Garcia-Hermoso et al., 2016), but also among adults with coronary artery disease or chronic heart failure (Haykowsky et al., 2013; Smart et al., 2013; Pattyn et al., 2014). As a supplementation, our study confirmed its effectiveness also in patients with type 2 diabetes, showing that INT increased VO_2max_ by 2.18 ml/kg/min compared with MICT and 6.38 ml/kg/min vs. NET. From a clinical perspective this is of significant importance, since every 1-metabolic equivalent increment equal to 3.5 ml/kg/min of O_2_ uptake is associated with an approximate 20% risk reduction in cardiovascular events or all-cause mortality among men with type 2 diabetes (Lyerly et al., 2008).

In addition, INT may help to optimize the glycemic control, showing reductions in HbA1c by 0.26% vs. MICT and 0.83% vs. NET, which were generally comparable to previous similar meta-analyses (Jelleyman et al., 2015; Liubaoerjijin et al., 2016). Yet the meta-analysis by Liubaoerjijin et al. included fewer studies on patients with type 2 diabetes than ours (Liubaoerjijin et al., 2016) and the one by Jelleyman et al. enrolled a mixed population (that is, patients with or at risk of type 2 diabetes) and did not restrict studies to solely RCTs (that is, analyzed controlled and uncontrolled studies) (Jelleyman et al., 2015). Noteworthy, the magnitude of HbA1c reduction associated with INT vs. NET for patients with type 2 diabetes is even slightly greater than that obtained from the low carbohydrate diet treatment (Meng et al., 2017) or antihyperglycaemic drug therapies like acarbose, empagliflozin, and DPP-4 inhibitors (Mearns et al., 2015), which is of significant clinical importance.

Notably, our study indicated that even in the condition of having similar energy expenditure, INT is more efficacious in improving cardiorespiratory fitness and glycemic control compared with MICT, suggesting that INT might be a valuable alternative to MICT in the general practice. Furthermore, our study showed that the improvements in cardiorespiratory fitness and glycemic control correlated with INT are independent of baseline anthropological parameters including age and BMI, disease severity, and fitness levels as assessed by HbA1c and VO_2max_, which were partly in agreement with previous findings (Jelleyman et al., 2015; Garcia-Hermoso et al., 2016). Moreover, they might provide some evidence that various subsets of patients with type 2 diabetes would gain health benefits from this form of exercise.

There is increasing evidence that weight loss, even with a modest magnitude, is strongly associated with improvements in glycemic control, lipid profiles, and blood pressure in patients with type 2 diabetes (Stevens et al., 2001; Espeland et al., 2013). Physical activity including regular aerobic exercise and unstructured daily movement has been recognized as a cornerstone in weight management (Colberg et al., 2010; Cai et al., 2016). However, our study did not provide adequate evidence that INT could reduce body weight compared with MICT nor NET. This is in line with the finding by Cassidy et al. stating that the role of INT in weight loss should not be overstated among patients with common metabolic disorders such as type 2 diabetes (Cassidy et al., 2017). Moreover, our study suggested that INT was more effective in lowering SBP rather than DBP compared with MICT or energy expenditure-matched MICT among patients with type 2 diabetes. This corresponds well with the finding from García-Hermoso et al. who observed similar results albeit in overweight and obese youth (Garcia-Hermoso et al., 2016). It is speculated that this might be partly explained by the evidence that INT could result in a higher reduction of sympathetic nervous activity (Hanada et al., 2011; Ciolac, 2012) as well as a larger improvement in endothelial function than MICT (Ciolac, 2012; Mitranun et al., 2014; Ramos et al., 2015), both factors are closely related to SBP control (Wallace et al., 2007; Flaa et al., 2008). Moreover, the INT-induced increases in shear stress might be another mechanical possibility (Adams et al., 2017). However, our study did not show that INT was sufficiently helpful for modifications in lipid profiles. These might be largely attributable to the small number of studies included with limited statistical power.

In addition to the efficacy, the safety of INT was evaluated by several individual studies (Terada et al., 2013; Alvarez et al., 2016; Cassidy et al., 2016; Maillard et al., 2016). Yet none of them reported potential harms like hypoglycemia, cardiovascular events, or sport-related injuries associated with INT in patients with type 2 diabetes. Meanwhile the adherence to INT was observed to be higher than 90% in average. However, one should be aware of the evidence that acute bout of exercise performed at vigorous- or high-intensity may lead to an increased risk of adverse responses (e.g., atrial tachycardia, myocardial infarction) among patients with cardiometabolic disease such as diabetes, at least transiently (Levinger et al., 2015). Given the suggestions from Levinger et al. (2015) and Haykowsky et al. (2013), and considering the position statement of the American Diabetes Association (Colberg et al., 2016), it is suggested that patients with type 2 diabetes who may wish to perform INT should require to be clinically stable, have been engaged at least in regular MICT, and be supervised or monitored at least initially.

### Limitations

Although this is the first study that provides comprehensive and quantitative analyses in exploring various cardiometabolic benefits of INT in patients with type 2 diabetes, it has certain limitations. First, as with other systematic reviews or meta-analyses focusing on INT (Beauchamp et al., 2010; Haykowsky et al., 2013; Garcia-Hermoso et al., 2016), our meta-analysis included studies that exhibited heterogeneity in INT protocols (Table 1). However, the trends for their effects were similar across studies in general, especially for cardiorespiratory fitness and glycemic control, which suggests that such differences in INT protocols are unlikely to substantially affect the health outcomes associated with INT. Moreover, the *I*^2^ values for assessing heterogeneity of the most outcomes were rather low, indicating that the variability in effect estimates was likely to be attributable to the chance of sampling error. Second, due to the limited comparable data from the small number of studies, our meta-analysis did not perform meta-regression analyses on exercise intensity or training duration. Yet it is noteworthy that almost all studies had INT at high- or marginally high-intensity. Third, although the enrolled patients who took medications were advised not to change the dosages of medications throughout the interventions, it cannot be ruled out that some of the medications, in particular like metformin, may blunt the full effect of exercise training on the outcomes of interest such as glycemic control (Boulé et al., 2011; Malin et al., 2012; Myette-Cote et al., 2016). Yet our study could not perform such an analysis restricted to patients who took metformin due to the inadequate information reported in the included studies. Finally, some publication bias was observed and we did not search gray literatures (e.g., doctoral theses or conference papers).

### Implications

This study provides evidence in support of the recommendation of INT for patients with type 2 diabetes (Colberg et al., 2016). Although Cassidy et al. stated that the desirable INT protocol might consist of high-intensity exercise intervals lasting 1–4 min for a total time of 10–20 min for each session, with 3 sessions per week (Cassidy et al., 2017), this statement seems to be partly subjective. As a result, the standardized INT protocols still remain to be explored and established (Colberg et al., 2016). Future studies with dose-response analyses that enroll large sample sizes with longer training periods over 16 weeks are warranted to determine the optimal and minimal training intensity, frequency, or duration of each interval. In addition, studies are also required to investigate whether replacing active recovery with passive one or using resistance exercise instead of aerobic exercise for recovery would exert comparable or superior health benefits. Furthermore, since most included studies were performed under supervision, it is relevant and requires investigation whether a similar effect would be observed in a “free-living” condition.

In conclusion, despite a limited number of studies, this meta-analysis shows that INT, which is at low-volume, conveys larger cardiometabolic benefits than MICT or NET for patients with type 2 diabetes, in particular for cardiorespiratory fitness and glycemic control. This indicates that INT is worth being recommended as an alternative approach for health promotion or in addition to the traditional aerobic exercise like MICT. However, its safety still remains to be explored and more studies with large sample sizes and long-term training periods are needed to determine the optimal INT protocols in the future.

## Author contributions

Conceived and conducted the study: SQ, XC, ZS, and US. Participated in data collection and data check: SQ and XC. Conducted data analysis and drafted the manuscript: SQ. Revised the manuscript: ZS, JS, MZ, and US. All authors approved the final version of the manuscript.

### Conflict of interest statement

The authors declare that the research was conducted in the absence of any commercial or financial relationships that could be construed as a potential conflict of interest.
